# Inflammation in children with cystic fibrosis: contribution of bacterial production of long-chain fatty acids

**DOI:** 10.1038/s41390-021-01419-4

**Published:** 2021-03-02

**Authors:** Erin Felton, Aszia Burrell, Hollis Chaney, Iman Sami, Anastassios C. Koumbourlis, Robert J. Freishtat, Keith A. Crandall, Andrea Hahn

**Affiliations:** 1 School of Medicine and Health Sciences, George Washington University, Washington, DC USA; 2 Center for Genetic Medicine Research, Children’s National Research Institute, Washington, DC USA; 3 Department of Pediatrics, George Washington University School of Medicine and Health Sciences, Washington, DC USA; 4 Division of Pulmonary and Sleep Medicine, Children’s National Hospital, Washington, DC USA; 5 Division of Emergency Medicine, Children’s National Hospital, Washington, DC USA; 6 Department of Biostatistics and Bioinformatics, Computational Biology Institute, Milken Institute School of Public Health, George Washington University, Washington, DC USA; 7 Division of Infectious Disease, Children’s National Hospital, Washington, DC USA

## Abstract

**Background:**

Cystic fibrosis (CF) affects >70,000 people worldwide, yet the microbiologic trigger for pulmonary exacerbations (PExs) remains unknown. The objective of this study was to identify changes in bacterial metabolic pathways associated with clinical status.

**Methods:**

Respiratory samples were collected at hospital admission for PEx, end of intravenous (IV) antibiotic treatment, and follow-up from 27 hospitalized children with CF. Bacterial DNA was extracted and shotgun DNA sequencing was performed. MetaPhlAn2 and HUMAnN2 were used to evaluate bacterial taxonomic and pathway relative abundance, while DESeq2 was used to evaluate differential abundance based on clinical status.

**Results:**

The mean age of study participants was 10 years; 85% received combination IV antibiotic therapy (beta-lactam plus a second agent). Long-chain fatty acid (LCFA) biosynthesis pathways were upregulated in follow-up samples compared to end of treatment: gondoate (*p* = 0.012), oleate (*p* = 0.048), palmitoleate (*p* = 0.043), and pathways of fatty acid elongation (*p* = 0.012). *Achromobacter xylosoxidans* and *Escherichia* sp. were also more prevalent in follow-up compared to PEx (*p* < 0.001).

**Conclusions:**

LCFAs may be associated with persistent infection of opportunistic pathogens. Future studies should more closely investigate the role of LCFA production by lung bacteria in the transition from baseline wellness to PEx in persons with CF.

**Impact:**

- Increased levels of LCFAs are found after IV antibiotic treatment in persons with CF.
- LCFAs have previously been associated with increased lung inflammation in asthma.
- This is the first report of LCFAs in the airway of persons with CF.
- This research provides support that bacterial production of LCFAs may be a contributor to inflammation in persons with CF.
- Future studies should evaluate LCFAs as predictors of future PExs.

## Introduction

Cystic fibrosis (CF) is an autosomal recessive disease affecting >30,000 people in the United States and 70,000 people worldwide.^1,2^ Lung disease in CF patients is characterized by chronic and intermittent acute lung infections. Sequela of these infections leads to an increase in respiratory symptoms accompanied by an acute decrease in lung function known as pulmonary exacerbations (PExs).^3^ *Staphylococcus aureus* and *Pseudomonas aeruginosa* remain the most common bacterial pathogens identified in routine culture and are thought to contribute to both lung inflammation and PEx.^2,4^ PEx occurs in patients with CF at all ages, and while advances in pulmonary and antibiotic therapy continue to extend the life of CF patients, PExs are still the main cause of morbidity, mortality, and decreased quality of life.^1,5^

For the past 15 years, 16S amplicon sequencing has been a commonly used culture-independent research technique to characterize the airway microbiome in CF patients.^6^ Through leveraging the phylogenetic data from 16S amplicon sequencing, we can characterize CF airway pathogens and now know that the CF lung microbial community is more diverse and complicated than previously thought.^7–9^ While some studies have shown a change in community structure to be associated with transitions between wellness, PEx, and following antibiotic treatment,^8,10^ others have not seen significant differences.^11^ Thus, simply sequencing to identify the bacteria present within the airway is likely insufficient to fully understand how and why bacteria within the airway can trigger PEx and how antibiotics affect the bacterial response.

While 16S amplicon sequencing can quantify the basic community composition and taxonomic profile of the CF lung, more recent studies for characterizing airway pathogens have used an unbiased whole-genome sequencing (WGS) approach.^12–14^ These early studies using next-generation sequencing (NGS) sequencing to characterize the airway have yielded greater insights into the species specificity of the lung microbiome, while little is still known about the functional genetics and antibiotic resistance mechanisms of the microbial populations and which bacterial functional pathways are associated with stages of CF lung disease. To better understand the relationship between the CF microbiome and changes in clinical status over time, including baseline wellness, PEx, and during and following antibiotic treatment, WGS coupled with metagenomic analyses of the CF airway can be used to investigate the bacterial functional profile, which aims to quantify the gene and metabolic pathway content contributed by known and uncharacterized microbiome community members.

The primary objective of this study was to identify changes in bacterial metabolic pathways associated with clinical status and intravenous (IV) antibiotic exposure (specifically, hospitalization for PEx, end of antibiotic treatment, and follow-up). The secondary objectives were to compare the metabolic changes with changes in bacterial species and antibiotic resistance. Our hypothesis is that significant differences in the bacterial metabolic profile are detectable between clinical status independent of differences associated with community composition. These findings will provide insight into the drivers associated with the transition between clinical status and identify opportunities for further study to improve management of PEx.

## Methods

### Study design

This was a single-center, prospective, longitudinal cohort study of persons ≤18 years of age with CF (positive genotype and/or sweat test >60) who were admitted to the hospital for treatment with IV antibiotics for a PEx. The study period occurred between May 2017 and December 2018. Institutional Board Review (IRB) approval from Children’s National Hospital IRB was obtained prior to beginning the study (Pro8047, 29 August 2016). The principles outlined in the Declaration of Helsinki were followed. Written informed consent was obtained from study participants who were 18 years of age and parental permission for those who were <18 years of age. Assent was obtained from participants 7–17 years of age. Spontaneous sputum and/or oropharyngeal (OP) swabs for microbiome studies were collected when patients were admitted for treatment with IV antibiotics (PEx), at the end of their antibiotic treatment course, and again at their next follow-up clinic appointment. PEx was defined using modified Fuch’s criteria, where (1) the patient was admitted for treatment with IV antibiotics and (2) the patient had at least one of the following 12 signs/symptoms that prompted the antibiotic therapy: change in sputum, new or increased hemoptysis, increased cough, increased dyspnea, fatigue/lethargy, fever >38 °C, anorexia/weight loss, sinus pain, change in sinus discharge, change in the physical examination of the chest, decrease in forced expiratory volume in one second (FEV1) by ≥10% the prior value, or radiographic changes consistent with pulmonary infection.^15^ Metadata were collected to correspond with each airway sample, including age, sex, race/ethnicity, cystic fibrosis transmembrane conductance regulator genotype, weight, height, medications, and results of pulmonary function tests (PFTs) and respiratory cultures.

### Respiratory sample processing

Collected respiratory samples were stored at 4 °C up to 72 h before sample processing. OP swab specimens were collected using an Eswab with Amies media (Copan). Spontaneous sputum samples were collected in a sterile specimen cup. Sputum samples were homogenized by mixing 1:1 (v/v) with sterile normal saline and dithiothreitol (Fisher Healthcare), vortexing, and heating in a 37 °C heated bead bath for 15 min, whereas the Eswab media were transferred directly. Samples were pelleted through centrifugation (12,000 × *g* for 10 min), supernatants were removed, and pellets were stored at −80 °C until DNA extraction.

### DNA extraction, quantity and quality determination, library prep, and sequencing

Pelleted bacterial cells were rapidly thawed and mixed with 1 mL of sterile phosphate-buffered saline. Bacterial DNA was extracted using a QIAamp DNA Microbiome kit (Qiagen), following the protocol as outlined by the company. Qubit (Thermo Fisher Scientific) was used for DNA quantification, and Bioanalyzer (Agilent) was used to verify DNA quality. Shotgun DNA sequencing was performed at the GW Genomics Core. DNA was diluted to a concentration of 10–20 ng/µL, and 5 µL were used for library construction using Nextera XT Library Prep kit (Illumina). Twelve to 15 libraries were sequenced per run on a NextSeq 500 (Illumina), using the Mid-Output 2 × 150 cycle kit, with an average of 12 million total sequences and 7.5 million aligned bacterial reads per sample.

### Bioinformatic approaches

FastQC and FlexBar were utilized to trim sequences prior to downstream analyses.^16^ KneadData were used to separate human from bacterial sequences.^17^ MetaPhlAn2 was used for taxonomic profiling and bacterial metabolic pathways were determined using the HUMANn2 workflow on Colonial One (GW).^18,19^ A count table of bacterial species was generated by using the arguments --tax_lev “s” and –t “rel_ab_w_read_stats” in our MetaPhlAn2 script. In our workflow of HUMANn2, we used MetaPhlAn2 for taxonomic profiling, ChocoPhlAn for a reference of functionally annotated species pangenomes, and Bowtie 2 for nucleotide-level pangenome mapping.^18,20,21^ Remaining unmapped reads were aligned against UniRef90 as its protein reference database using DIAMOND.^22,23^ *MetaCyc* was utilized for pathway collection.^24^ Unstratified (i.e., unlinked from associated bacterial taxa) pathway abundance output tables were generated using the utility script “humann2_split_stratified_table” and used in subsequent analysis. AmrPlusPlus was used to align filtered and trimmed bacterial sequences against antibiotic resistance genes in Galaxy to generate count tables of class-level resistance.^25^ Observed species/pathways and taxonomy/pathway tables were imported into Rstudio v3.6.1 for subsequent analyses using the packages *DESeq2* v.1.24.0, *ggplot2* v3.2.0, *phyloseq* v.1.28.0, and *vegan* v.2.5-6.^26^ Alpha diversity was measured by the number of observed bacterial species, Shannon index, and the inverse Simpson’s index using Explicet v.2.10.5.^27^

### Statistical analyses

The normality of continuous data was tested using Shapiro–Wilk in STATA/IC (v.15.1). As FEV1 (*p* = 0.006), the number of observed bacterial species (*p* = 0.046), and the inverse Simpson’s index (*p* < 0.001) were skewed, square, square root, and log transformations were applied to normalize the distributions, respectively. Generalized linear models, using a Gaussian variance function and an identity link function, were then used for comparison of alpha-diversity measures between groups, controlling for repeated patient measures. Permutational analysis of variance was performed in Rstudio using the *adonis* function, using Bray distance matrices and *strata* to control for repeated samples obtained from each study participant.

### Sensitivity analysis

Twenty-one of the sequenced samples used in subsequent analyses were from sputum, while 50 were from OP swabs. We collected and sequenced three sputum/OP swab pairs to understand the variability introduced by collecting different respiratory sample types within our cohort. We evaluated for differences in richness and alpha diversity and dissimilarity in community composition between the sputum/OP swab pairs. No significant differences were noted in alpha diversity between the sputum and OP samples (Supplementary Table 1). When evaluating community composition, the relative abundance of bacterial taxa between the paired sputum and swab samples were significantly correlated with each other (*R*^2^ range 0.779–0.968, all *p* < 0.001, Supplementary Fig. 1). In addition, the high similarity between the sputum/swab pairs was demonstrated using the Morisita–Horn index (range 0.861–0.908), and the Bray–Curtis distances were not significantly dissimilar (Supplementary Fig. 2). Therefore, we felt confident in proceeding with our study objectives.

## Results

### Study participants and clinical parameters

Twenty-seven study participants were followed from PEx onset, defined as hospital admission for IV antibiotic therapy, through antibiotic treatment and their next follow-up in pulmonary clinic (Table 1). Most participants were male (56%) and Caucasian (81%), with a mean age of 10 years. Most participants were not receiving any suppressive antibiotic and anti-inflammatory therapy at baseline (63%). At the time of PEx, a mean of 3.3 (SD 1.2) of the 12 Fuch’s criteria were reported per study participant. Most participants reported an increased cough (85%), with the next most commonly reported symptom being a change in sputum production (44%) followed by dyspnea (30%). The most commons signs of PEx were a change in the physical exam of their chest (41%), radiographic changes consistent with infection (37%), and a decrease in their FEV1 of ≥10% (37%). The mean percent predicted FEV1 at exacerbation onset was 81.7%, while the mean percent predicted forced vital capacity (FVC) was 87.7% and the mean percent predicted forced expiratory flow 25–75 (FEF25–75) was 72.4%. The most common respiratory pathogens at PEx onset were *P. aeruginosa* and *S. aureus*, although 41% of participants only grew normal respiratory flora (a designation by our microbiology laboratory for bacteria commonly found in the upper airway, including alpha hemolytic *Streptococci* and other oral anaerobes). Eighty-five percent of study participants received combination antibiotic therapy (beta-lactam plus another drug class). For the participants old enough to perform PFTs, the best FEV1 in the 6 months preceding their PEx was 96.4% (SD 16.7). The majority of study participants had an early disease stage with an FEV1 > 70% (85%, *n* = 23), while one study participant was considered to be at an intermediate disease stage (FEV1 between 40 and 70%).^28^ Three study participants were too young to perform PFTs. Pulmonary function was improved at the end of antibiotic treatment and follow-up compared to PEx, although not significantly (FEV1 *p* = 0.303, FVC *p* = 0.099, and FEF25–75 *p* = 0.267).

**Table 1:** Demographics and clinical parameters of study participants

Clinical parameters	*n* = 27
Sex (male:female)	15:12
Age at PEx (mean years, range)	10 (1–18)
Race (*n*, %)	
Caucasian	22 (81%)
African American	4 (15%)
Unknown	1 (4%)
Ethnicity (*n*, %)	
Hispanic/Latino	10 (37%)
Not Hispanic/Latino	17 (63%)
CF genotype (*n*, %)	
F508del homozygous	12 (44%)
F508del heterozygous	11 (41%)
Other	4 (15%)
CFTR modulator use^a^ (*n*, % yes)	7 (26%)
History of prior *Pseudomonas aeruginosa* infection (*n*, % yes)	18 (67%)
Prior use of suppressive antibiotic/anti-inflammatory therapy (*n*, %)	
Inhaled therapy	6 (22%)
Oral and inhaled therapy	4 (15%)
No therapy	17 (63%)
Oral antibiotics in the 30 days preceding initiation of IV therapy (*n*, % yes)	11 (41%)
Signs/symptoms at PEx onset (*n*, %)	
Cough	23 (95%)
Sputum production	12 (44%)
Dyspnea	8 (30%)
Fever	6 (22%)
Fatigue	4 (15%)
Change in sinus discharge	2 (7%)
Sinus pain	1 (4%)
Anorexia	1 (4%)
Hemoptysis	1 (4%)
10% decrease in FEV1	10 (37%)
10% decrease in FEF25–75	11 (41%)
Change in chest exam (e.g., rales)	11 (41%)
New CXR findings	10 (37%)
Culture results at PEx onset (*n*, %)^b^	
* Pseudomonas aeruginosa* (rough strain)	6 (22%)
* Pseudomonas aeruginosa* (mucoid strain)	4 (15%)
* Staphylococcus aureus* (MSSA)	4 (15%)
* Staphylococcus aureus* (MRSA)	3 (11%)
* Moraxella catarrhalis*	1 (4%)
* Achromobacter xylosoxidans*	1 (4%)
*Stenotrophomonas maltophilia*	1 (4%)
* Haemophilus influenza*	1 (4%)
Unidentified Gram-negative rod	1 (4%)
Only normal respiratory flora	11 (41%)
Beta-lactam antibiotics received for PEx (*n*, %)^c^	
Ceftazidime	15 (56%)
Piperacillin-tazobactam	6 (22%)
Cefepime	5 (19%)
Meropenem	4 (15%)
Ceftriaxone	2 (7%)
Other antibiotics received for PEx (*n*, %)^d^	
Tobramycin	17 (63%)
Vancomycin	5 (19%)
Aztreonam	1 (4%)
Ciprofloxacin	1 (4%)
Duration of antibiotic treatment (mean days ± SD)	15.9 ± 4.7
Days between date of hospitalization and date of follow-up (mean days ± SD)	65.4 ± 36
FEV1 % predicted (mean ± SD)	
PEx (*n* = 23)	81.7 ± 19.4
End of antibiotic treatment (*n* = 24)	91 ± 17.7
Follow-up (*n* = 21)	93 ± 18.2
FVC % predicted (mean ± SD)	
PEx (*n* = 23)	87.7 ± 16.6
End of antibiotic treatment (*n* = 24)	95.5 ± 13
Follow-up (*n* = 21)	96.7 ± 14.4
FEF25–75% predicted (mean ± SD)	
PEx (*n* = 23)	72.4 ± 28.5
End of antibiotic treatment (*n* = 24)	90.1 ± 37
Follow-up (*n* = 21)	88.8 ± 32

*PEx* pulmonary exacerbation, *CFTR* cystic fibrosis transmembrane conductance regulator.

^a^Ivacaftor-lumacaftor was the only CFTR modulator used in this study cohort.

^b^Some cultures had multiple bacteria present: *P. aeruginosa* (rough strain) + *S. maltophilia* (*n* = 1), MRSA + unidentified Gram-negative rod (*n* = 1), MSSA + *H. influenzae* (*n* = 1), MSSA + MRSA + *P. aeruginosa* (rough strain) (*n* = 1), and *P. aeruginosa* (rough strain) + *P. aeruginosa* (mucoid strain) (*n* = 2).

^c^Some beta-lactams were switched during the treatment course: piperacillin-tazobactam to meropenem (*n* = 1), piperacillin-tazobactam to ceftazidime (*n* = 1), ceftazidime to piperacillin-tazobactam (*n* = 1), ceftazidime to cefepime (*n* = 1), and ceftazidime to meropenem to cefepime (*n* = 1).

^d^Only one agent was added to the backbone beta-lactam, except in one instance where both vancomycin + tobramycin were given.

### Sequencing results

Sequencing data were obtained for 71 of 81 study time points (three missed collection; seven failed sequencing). The mean number of sequences was 9.7 million (range 668K–21 million), and the mean number of aligned sequences was 7.9 million (range 12K–18.9 million). Four Zymo controls were run with the study samples and the mean relative abundance was significantly concordant with the expected relative abundance of the defined microbial community consisting of eight bacteria and two yeast (*R*^2^ = 0.71, *p* = 0.002; Supplementary Table 2).

### Bacterial species richness, relative abundance, and alpha-diversity measures

**Fig. 1:**
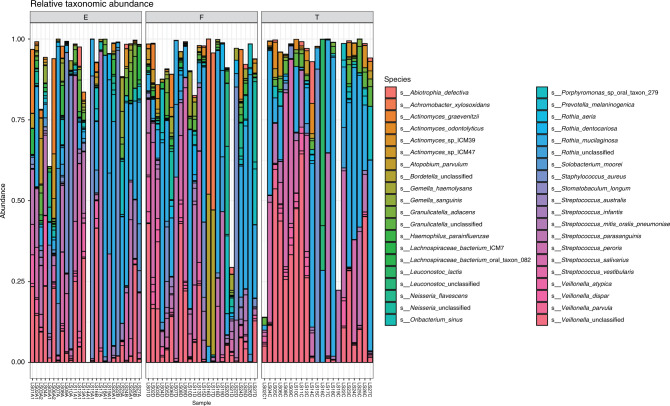
Relative taxonomic abundance. The 60 most abundant bacterial species of 195 bacterial species identified are shown. E exacerbation, F follow-up, T treatment.

**Fig. 2:**
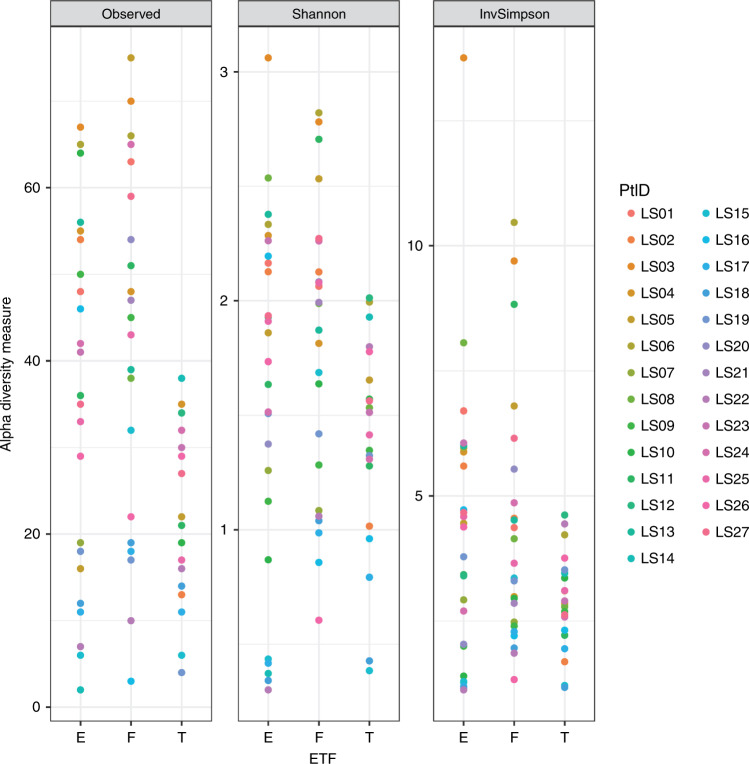
Richness and alpha-diversity measures. Number of observed species, Shannon index, and inverse Simpson Index are included. The *y*-axis gives the measurement, while the *x*-axis is organized by clinical status. E exacerbation, T treatment, F follow-up. Samples are color coded according to the study participant ID number.

**Fig. 3:**
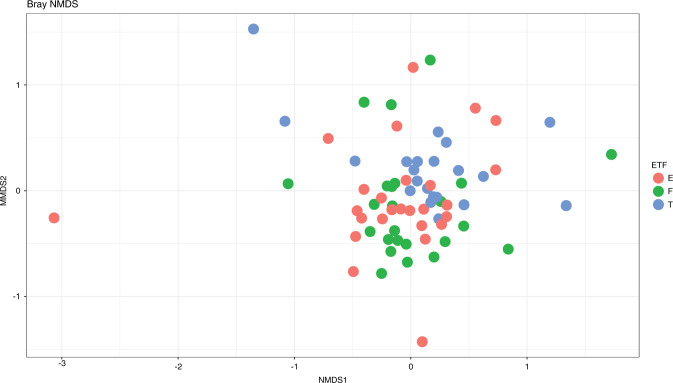
Bray–Curtis non-metric multidimensional scaling plot. No significant difference in overall community composition was identified between changes in clinical status using permutational analysis of variance (PERMANOVA), controlling for repeated samples in the same participant (*R*^2^ = 0.031, *p* = 0.062). E exacerbation, T treatment, F follow-up.

One hundred and ninety-five bacterial species were identified across all samples. There was an average of 34 bacterial species identified per sample (SD 19, range 2–75), and the relative abundance of the different bacterial species by sample is shown in Fig. 1. Across the whole cohort of samples, 13 species had a relative abundance of >1% and accounted for 81.6% of all reads. These included the following: *Rothia mucilaginosa* (26%), *Veillonella*_unclassified (16.5%), *Streptococcus salivarius* (10.9%), *Streptococcus parasanguinis* (9.5%), *Staphylococcus aureus* (4.9%), *Porphyromonas* sp. (oral taxon 279, 2.4%), *Actinomyces graevenitzii* (2.2%), *Granulicatella*_unclassified (1.9%), *Leuconostoc lactis* (1.6%), *Neisseria*_unclassified (1.6%), *Veillonella atypica* (1.5%), *Veillonella dispar* (1.5%), and *Rothia*_unclassified (1.2%). Alpha diversity was also measured to evaluate the balance of species within the samples and their relative abundance (Fig. 2). The average Shannon diversity index across all samples was 2.30 (SD 0.94, range 0.43–4.42), and the average inverse Simpson index was 3.81 (SD 2.33, range 1.12–13.76). While bacterial species richness and alpha diversity was decreased at the end of antibiotic treatment compared to exacerbation onset and follow-up while controlling for repeated samples obtained from the same participant, only richness was statistically significant (*p* = 0.027, Table 2). We also did not appreciate any differences in overall community composition based on clinical status, calculating Bray–Curtis dissimilarity while controlling for repeated samples collected from the same participant (Fig. 3).

**Table 2:** Comparison of richness and alpha diversity across clinical states

Clinical state	Mean	Standard error	*P* value
Number of observed bacterial species			
Exacerbation^a/b^	36	3.3	0.027^a^
End of antibiotic treatment^c^	23	3.7	
Follow-up^d^	41	3.4	
Shannon diversity index			
Exacerbation^a/b^	2.31	0.18	0.260^b^
End of antibiotic treatment^c^	2.01	0.20	
Follow-up^d^	2.58	0.18	
Inverse Simpson index			
Exacerbation^a/b^	4.14	0.43	0.226^c^
End of antibiotic treatment^c^	2.96	0.48	
Follow-up^d^	4.28	0.45	

^a^Generalized linear model following square root transform, controlling for repeated samples.

^b^Generalized linear model, controlling for repeated samples.

^c^Generalized linear model following log transform, controlling for repeated samples.

### Bacterial metabolic pathways across changes in clinical status

**Fig. 4:**
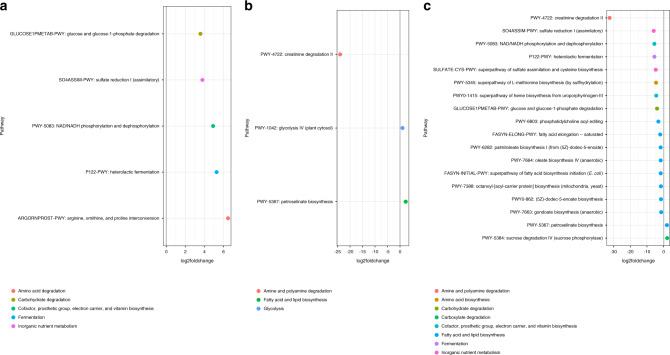
Differential abundance of unstratified bacterial metabolic pathways between changes in clinical status. **a** Differential abundance of bacterial pathways higher in exacerbation onset samples (right). **b** Differential abundance of bacterial pathways higher in exacerbation onset (right) and higher in follow-up samples (left). **c** Differential abundance of bacterial pathways higher in the end of antibiotic treatment samples (right) and higher in follow-up samples (left). All adjusted *p* values are <0.05.

Generally, we found that pathways related to bacterial metabolic pathways of biosynthesis, degradation/utilization, and fermentation were differentially present based on clinical status (Fig. 4). Only a few degradation/utilization and fermentation pathways and a biosynthesis pathway were more abundant in PEx samples when compared to the end of treatment samples (Fig. 4a). Similarly, only a few pathways were more abundant in follow-up samples compared to PEx samples (a glycolysis and a fatty acid biosynthesis pathway), while an amine degradation pathway was more abundant in follow-up samples (Fig. 4b). However, we identified many fatty acid and lipid biosynthesis pathways that were present with a higher abundance in follow-up samples compared to the end of treatment samples, especially related to the synthesis and elongation of long-chain fatty acids (LCFAs) (Fig. 4c). These include phosphatidylcholine acyl editing (log2fold change −3.2, *p* < 0.001), fatty acid elongation (log2fold change −2.1, *p* < 0.001), palmitoleate biosynthesis (log2fold change −1.8, *p* < 0.001), oleate biosynthesis (log2fold change −1.8, *p* < 0.001), superpathway of fatty acid biosynthesis initiation (log2fold change −1.8, *p* < 0.001), octanoyl biosynthesis (log2fold change −1.6, *p* = 0.002), dodec-5-enoate biosynthesis (log2fold change −1.6, *p* = 0.002), and gondoate biosynthesis (log2fold change −1.5, *p* < 0.001). Sulfate assimilation/cysteine synthesis (log2fold change −4.7, *p* < 0.001) and sulfate reduction pathways (log2fold change −6, *p* < 0.001) were also more abundant in follow-up samples when compared to the end of antibiotic treatment samples (Fig. 4c). Other pathways differentially abundant between end of antibiotic treatment samples compared to follow-up samples included biosynthesis, degradation, and fermentation pathways (Fig. 4c).

### Differential abundance of bacterial species across changes in clinical status

**Fig. 5:**
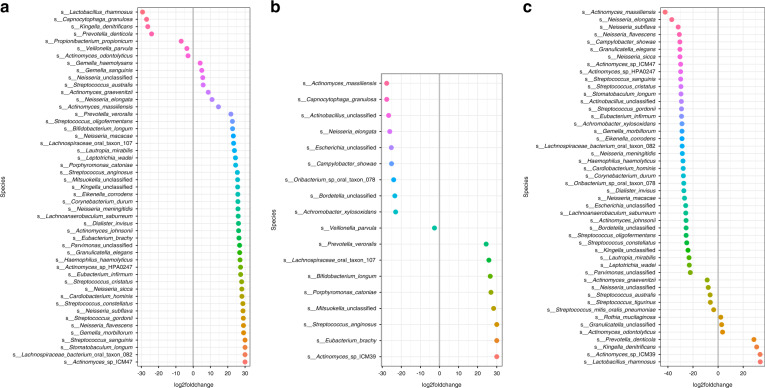
Differential abundance of bacterial species between changes in clinical status. **a** Differential abundance of bacterial species higher in exacerbation onset samples (right) and higher in end of antibiotic treatment samples (left). **b** Differential abundance of bacterial species higher in exacerbation onset (right) and higher in follow-up samples (left). **c** Differential abundance of bacterial species higher in end of antibiotic treatment samples (right) and higher in follow-up samples (left). All adjusted *p* values are <0.05.

Many bacterial species were noted to be differential abundant based on clinical status (Fig. 5). When comparing PEx versus end of treatment samples, we found that 42 bacterial species were significantly more likely to be present in PEx, while seven bacterial species were more likely to be present at the end of treatment (all adjusted *p* value < 0.05 and log2fold > |2|, Fig. 5a). Interestingly, the majority of the species more likely to be present at PEx included many “normal” respiratory flora, including several *Gemella* sp., *Neisseria* sp., and *Streptococcus* sp. When comparing PEx versus follow-up samples, we found that eight bacterial species were more likely to be present in PEx, while ten bacterial species were more likely to be present in follow-up (all adjusted *p* value <0.05 and log2fold > |2|, Fig. 5b). Again, most of the bacteria that were differentially abundant would be considered “normal” flora, with the exception of *Achromobacter xylosoxidans*, which was more abundant in follow-up samples (log2fold change of −23, adjusted *p* < 0.001) and *Escherichia*_unclassified (log2fold change of −25, adjusted *p* < 0.001). Lastly, when comparing end of treatment versus follow-up samples, we found that seven bacterial species were more likely to be present at the end of treatment, while 41 bacterial species were more likely to be present at follow-up (all adjusted *p* value < 0.05 and log2fold > |2|, Fig. 5c). Again, most of these bacterial species would be considered normal respiratory flora. However, *A. xylosoxidans* and *Escherichia*_unclassified remained as significantly more likely to be present in follow-up samples (log2fold change of −29 and −26, adjusted *p* < 0.001, respectively).

### Antibiotic class-level resistance across changes in clinical status

**Fig. 6:**
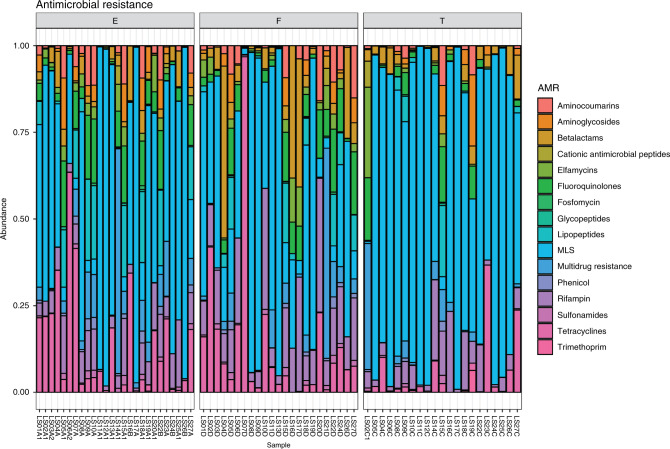
Relative abundance of antibiotic class resistance. MLS macrolides, lincosamides, and streptogramin A and B drugs.

The mean number of sequences that aligned against antibiotic resistance genes was 16K (range 0–242K). The most common antibiotic class resistance among all samples was macrolides, lincosamides, and streptogramin A and B drugs (MLS); the relative abundance of all antibiotic resistance classes is shown in Fig. 6. When exploring between changes in clinical status, we found that beta-lactam class resistance was significantly more likely to be present in treatment samples compared to PEx samples (log2fold change −1.7, adjusted *p* = 0.017). In addition, treatment samples were more likely to have MLS class resistance compared to follow-up samples (log2fold change 2.3, adjusted *p* = 0.025), while follow-up samples were more likely to have fluoroquinolone resistance compared to treatment samples (log2fold change −1, adjusted *p* = 0.025). There were no significant differences between PEx and follow-up samples.

## Discussion

In this study, we used NGS sequencing to gain insight into the functional profiles (metabolic pathways) of the CF lung community across changes in clinical status. Pathways related to biosynthesis, degradation/utilization, and fermentation, which contribute to pathogenicity and virulence, were found to be differentially abundant between respiratory samples obtained at PEx, the end of antibiotic treatment, and in follow-up. Sulfate assimilation/cysteine synthesis and sulfate reduction pathways were more prevalent in the follow-up samples when compared to the end of antibiotic treatment samples. Sulfate and cysteine metabolism play a role in extracellular phospholipase activity and flagellum-mediated surface motility in *Serratia* sp., both mechanisms of pathogenesis and virulence, and may be seen in other bacteria as well.^29^ Sulfate assimilation has also been shown to enhance microbial growth for *Escherichia coli* and other bacterial species.^30^ As these mechanisms were more predominant in the follow-up samples, it is likely that these may be related to the *Escherichia* sp. and allow for sustained growth after antibiotic exposure.

Perhaps, the most interesting finding was that multiple LCFA biosynthesis pathways and fatty acid elongation pathways were differentially abundant in follow-up samples compared to treatment samples. The LCFA biosynthesis pathways include dodec-5-enoate, gondoate, octanoyl-[*acp*], oleate, and palmitoleate. Interestingly, we did not find any pathways specific to short-chain fatty acids (SCFA) to be differentially abundant at any time point. Free fatty acids can exert physiologic or pathophysiologic effects by acting on free fatty acid receptors in tissue. Gut-derived SCFAs have been studied extensively as yielding a protective effect on inflammation, leading to protection in inflammatory diseases across multiple organ systems. In addition, deficiencies in gut-derived SFCAs have been shown to be associated with inflammatory illnesses.^31–34^ In contrast to SCFAs, LCFAs acting on their receptors can have a pathogenic rather than protective effect in certain metabolic diseases.^35^ LCFA may not only have a role in metabolic diseases but inflammatory disease as well. As obesity is a major risk factor for asthma, current research has looked into the effect of free LCFAs on airway remodeling and lung inflammation by acting on long-chain FFA receptors in the lung.^36,37^ LCFAs have been shown to induce bronchial cell proliferation, airway remodeling, and airway smooth muscle contraction—all important factors in lung inflammation and exacerbation.^38–40^ While most of this research pertains to fatty acids introduced through diet or in the lab, we can speculate that LCFAs produced by lung pathogens in the CF airway can have a physiologic effect like gut-derived bacterial SCFA. But instead of the protective effect of SCFA, the LCFAs produced by CF lung bacteria may have an effect on the level of inflammation in the lung. As these LCFA pathways were mostly upregulated in the follow-up samples sometime after antibiotic treatment, this could give us insight into the steady-state metabolic environment of the CF lung and how it relates to inflammation. These insights along with future longitudinal studies may help us further characterize the lung after recovery from PEx and what might make the CF lung prone to future PEx.

We also evaluated the change in bacterial species related to change in clinical status While *P. aeruginosa* has commonly been shown to be a dominant part of the CF airway and often plays a significant role in the CF lung and CF PEx, it was found to be of low relative abundance in our microbiome analysis study even though it was the most common pathogen identified in CF respiratory culture.^8,41,42^ One possible reason for this may be the methods by which CF respiratory cultures are performed in microbiology laboratories, which prioritize the growth of Gram-positive and Gram-negative aerobic organisms.^43^ Another possibility is the epidemiology of *P. aeruginosa* in the CF population. *Pseudomonas aeruginosa* is much more common in adolescents and older adults and tends to overtake the microbiome in more advanced lung disease.^9,11,43,44^ Our study population was younger with early disease stage, and so *P. aeruginosa* should not be expected to be a dominant part of their overall bacterial community. Our study did find *Gemella* sp. to be more abundant in PEx compared to end of treatment samples, which corroborates other published studies^8^ and suggests that *Gemella* sp. may contribute to PEx in children with CF. Our findings that *A. xylosoxidans* and *Escherichia* sp. were more common in follow-up samples may be due in part to their antibiotic resistance profiles. *Achromobacter xylosoxidans* is an opportunistic pathogen in persons with CF^45^ that frequently has antibiotic resistance mechanisms against beta-lactams and aminoglycosides,^13,46,47^ which was the most commonly used antibiotic combination in our study. *Escherichia coli* has also been reported to cause persistent infection in children and adults with CF,^48,49^ which may be why we identified *Escherichia* sp. more often in follow-up samples.

Lastly, we sought to identify changes in antibiotic resistance associated with changes in clinical status in our cohort, as antibiotic exposure is known to drive antibiotic resistance. While only a few of our study participants were receiving azithromycin therapy as part of their routine care, macrolide/lincosamide/streptogramin class resistance was the most commonly identified across the cohort. Treatment samples were also more likely to have macrolide/lincosamide/streptogramin resistance, even though neither azithromycin nor clindamycin was given during the inpatient antibiotic treatment course. Macrolide resistance in *Streptococcus* isolates from persons with CF has previously been reported to be as high as 50–75%, and clindamycin resistance has been reported at 25–50%.^50–52^ As >20% of the total relative abundance of our cohort was *Streptococcus* sp., it is likely that this species contributed to our antibiotic class resistance findings. We also found beta-lactam resistance to be more common in treatment samples compared to PEx samples, which was likely related to antibiotic selection pressure leading to a reduction of beta-lactam susceptible bacteria. Lastly, we found fluoroquinolone resistance to be more prevalent in follow-up samples compared to treatment samples. Interestingly, *A. xylosoxidans* frequently carry resistance genes against fluoroquinolones, and so this finding may be related to this bacteria also being more prevalent in follow-up samples.^13,47^

This was a monocentric study with a smaller number of study participants, so our findings may not be generalizable to a larger population of persons with CF. Another limitation to this study was the use of both OP and sputum respiratory specimens, as this may have increased our findings of traditionally considered “upper airway” flora in our samples.^53^ In addition, some studies have suggested that OP swab samples may underrepresent the presence of more traditional CF pathogens, such as *P. aeruginosa*.^54^ However, in our cohort, we found similarities between paired collections of different sample types in our sensitivity analysis. Future studies will collect samples from spontaneous expectorators to remove this potential bias and validate our findings. Our follow-up time point of collection was based on visits requested by the clinical team. While this typically was requested 1 month after hospitalization, there was a large variability in when the children returned to the clinic and thus in the collection of this sample. Future studies should include a research visit to ensure more consistency in the timing of the follow-up sample collection. We also did not perform a sub-analysis based on disease severity, but the majority of participants were at early disease stage or too young to complete spirometry. Future studies should include also include study participants with intermediate and advanced stage disease to assess the generalizability of our study findings. Lastly, we did not perform a sub-analysis based on infection pathogen, but given the small numbers our interpretation would have been limited.

In summary, our study findings suggest that following an IV antibiotic treatment course, LCFAs may be associated with continued inflammation due to opportunistic and persistent pathogens. Future longitudinal studies will allow for a better understanding of how the bacterial function profile after antibiotic treatment could be used to predict risk for subsequent PEx. In addition, future studies should more closely investigate the role of LCFA production by lung bacteria in the transition from baseline wellness to PEx in persons with CF.

## Supplementary information

Supplementary Information

## Data Availability

The sequence dataset supporting the conclusions of this article is available in the NCBI SRA repository under BioProject PRJNA615628. The batch and R scripts used for bioinformatic analyses have been uploaded to GitHub (github.com/alhahn/CF_functional_profiling).
