# Supplementary material for: Inflammation in children with cystic fibrosis: contribution of bacterial production of long-chain fatty acids
Source: Pediatr Res. 2021 Mar 2;90(1):99–108. doi: 10.1038/s41390-021-01419-4 (PMC8370878; doi:10.1038/s41390-021-01419-4)
Supplement: Supplementary file 1 — Supplementary Information [file 41390_2021_1419_MOESM1_ESM.pdf]

Bacterial functional profiling of the cystic fibrosis airway across clinical states

Erin Felton BA, Aszia Burrell BS, Hollis Chaney MD, Iman Sami MD, Anastassios C. Koumbourlis MD, MPH, Robert J. Freishtat MD, MPH, Keith A. Crandall PhD, and Andrea Hahn MD, MS

Supplemental Table 1. Alpha diversity measures of paired sputum and oropharyngeal swab samples

	Sputum (n=3)	Oropharyngeal swab (n=3)	P-value
Observed species (mean, standard deviation (SD))	67.7 (16.7)	57.7 (11.9)	0.551
Shannon diversity (mean, SD)	3.37 (0.61)	3.09 (1.37)	0.808
Inverse Simpson index (mean, SD)	5.61 (2.3)	6.68 (5.21)	0.808

Supplemental Table 2. Relative abundance of bacterial community controls

Bacteria/yeast	Expected microbial community (%)	Zymo_C1 (%)	Zymo_C2 (%)	Zymo_C3 (%)	Zymo_C4 (%)	Mean (%)
P. aeruginosa	12	19.43	9.07	9.79	8.43	11.68
E. coli	12	9.25	7.43	8.49	4.14	7.33
S. enterica	12	10.44	10.97	11.54	9.22	10.55
L. fermentum	12	24.72	20.12	20.16	13.19	19.55
E. faecalis	12	8.11	12.91	13.92	16.34	12.82
S. aureus	12	3.42	12.93	11.57	21.29	12.30
L. monocytogenes	12	6.26	11.10	11.72	13.18	10.56
B. subtilis	12	10.18	10.55	10.97	9.19	10.22
S. cerevisiae	2	0.13	0.57	0.46	0.68	0.46
C. neoformans	2	0.12	0.27	0.23	0.14	0.19
Other	0	7.94	4.08	1.14	4.18	4.34

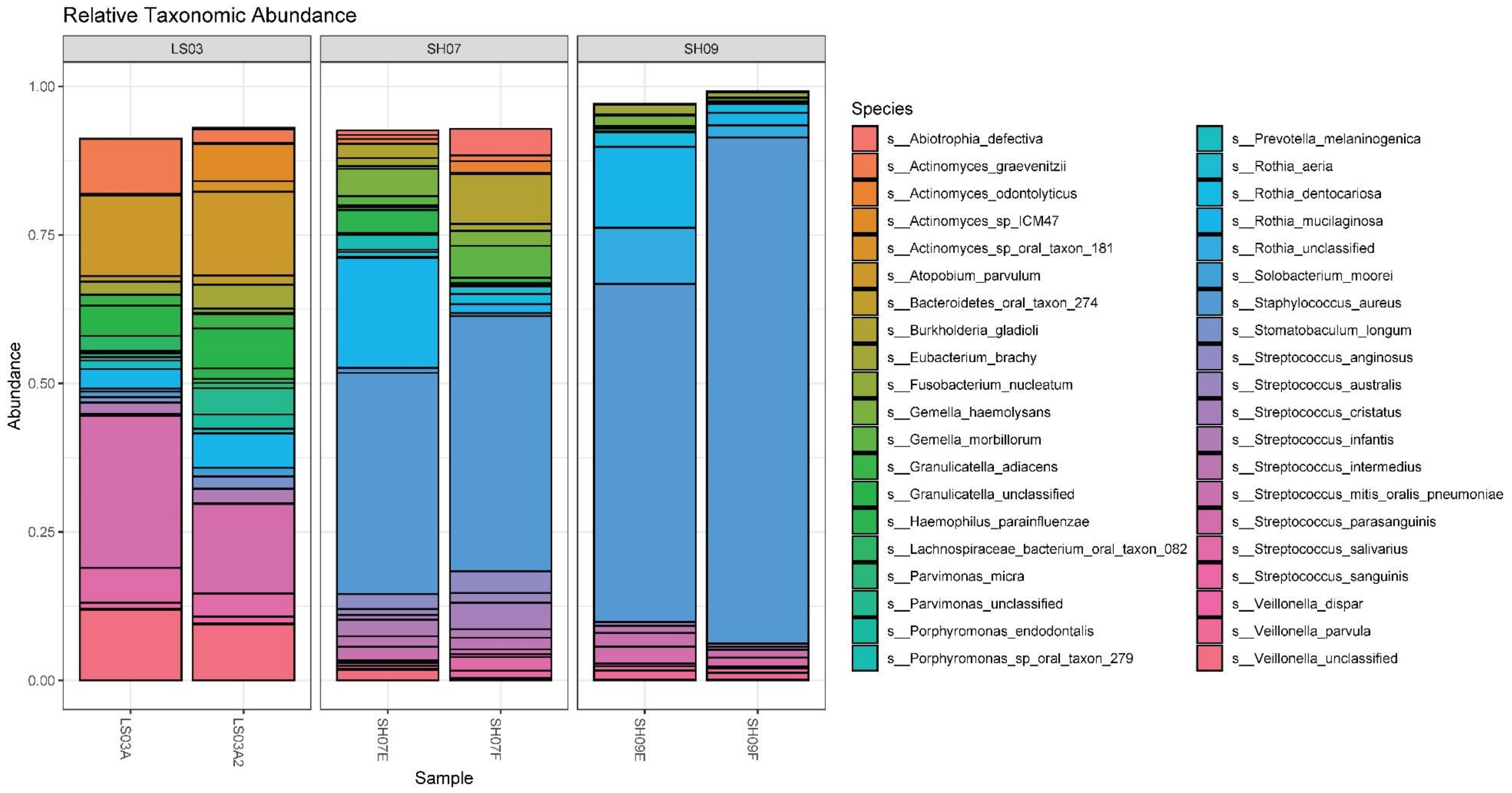

Supplemental Figure 1. Relative abundance of bacterial taxa of paired sputum and oropharyngeal swab samples. The top 40 taxa are shown.

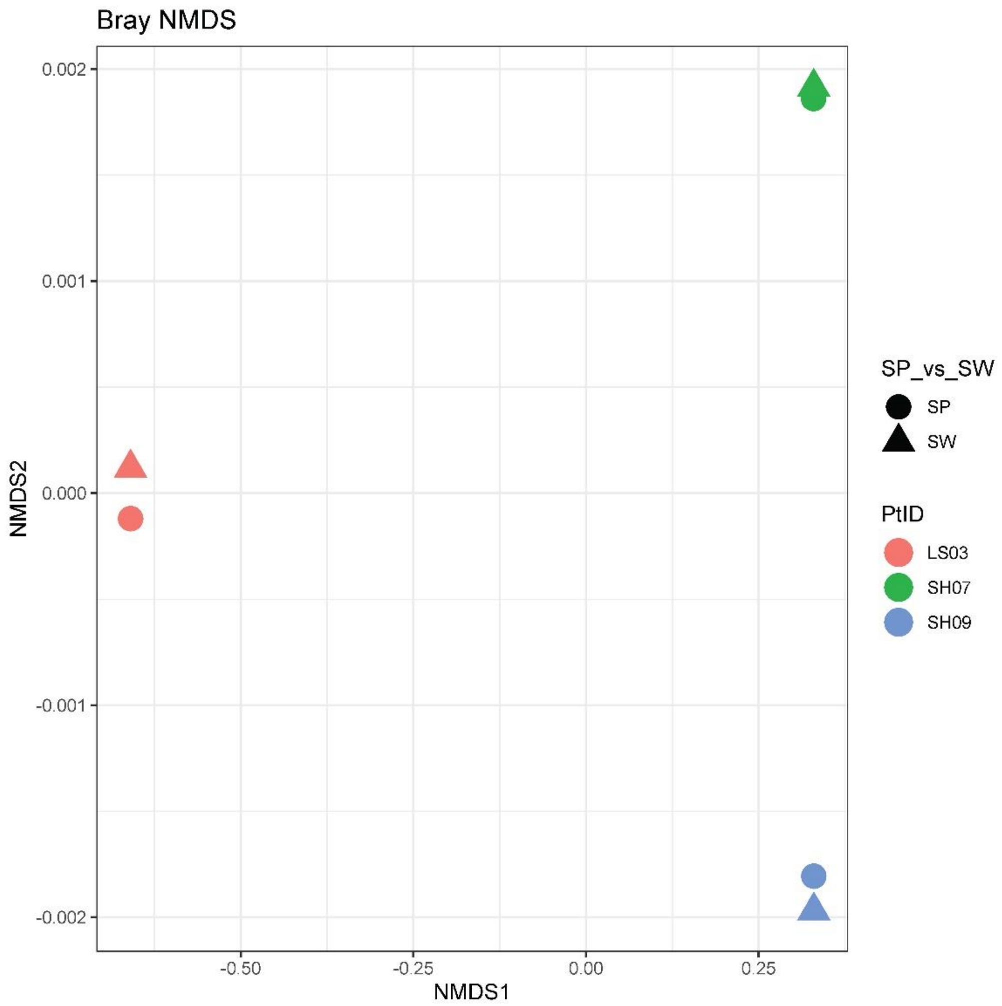

Supplemental Figure 2. Bray-curtis non-metric multi-dimensional scaling plot of paired sputum and oropharyngeal swab samples. No significant difference in overall community composition was identified between paired samples using permutational analysis of variance (PERMANOVA), controlling for paired samples using the strata function (R^2=0.137, p=0.250). SP, sputum, SW, swab.
